# Roles of Single Nucleotide Polymorphisms of *C3* Gene in Patients with Coronary Artery Disease

**DOI:** 10.31083/j.rcm2504147

**Published:** 2024-04-18

**Authors:** Shajidan Abudureyimu, Chunhui He, Dilihumaer Abulaiti, Wei Xie, Halisha Airikenjiang, Haitang Qiu, Mengjia Liu, Yan Cao, Hui Li, Jian Zhang, Ying Gao

**Affiliations:** ^1^Department of Comprehensive Internal Medicine, The First Affiliated Hospital of Xinjiang Medical University, 830011 Urumqi, Xinjiang, China; ^2^Heart Failure Center, State Key Laboratory of Cardiovascular Disease, Fuwai Hospital, National Center for Cardiovascular Diseases, Chinese Academy of Medical Sciences & Peking Union Medical College (CAMS & PUMC), 100010 Beijing, China; ^3^Department of Cardiology, Xinjiang Production and Construction Corps Hospital, 830011 Urumqi, Xinjiang, China; ^4^Department of Hepatobiliary and Pancreatic Surgery, Affiliated Cancer Hospital of Xinjiang Medical University, 830000 Urumqi, Xinjiang, China; ^5^Key Laboratory of Clinical Research for Cardiovascular Medications, National Health Committee, 100010 Beijing, China

**Keywords:** coronary artery disease, complement C3, gene polymorphism

## Abstract

**Background:**

This study aims to investigate the association between nine tag single nucleotide polymorphisms (SNPs) in the *C3* gene locus and the risk of coronary artery disease (CAD) as well as lipid levels in the Chinese population, and to further explore the interactions between SNPs and environmental factors that may be associated with CAD risk.

**Methods:**

A case-control study was conducted to investigate the association between CAD and *C3* gene polymorphisms in a hospital setting. The study consisted of 944 CAD patients with a mean age of 55.97 ± 10.182 years and 897 non-CAD controls with a mean age of 55.94 ± 9.162 years. There were 565 males and 288 females in the CAD group and 583 males and 314 females in the control group. TagSNPs in the *C3* gene were identified by employing the improved multiplex ligation detection reaction (iMLDR) technique, and multifactor dimensionality reduction (MDR) analysis was utilized to investigate the *C3* gene–environment and gene–gene interactions in relation to the risk of CAD.

**Results:**

Results of the polymorphism study indicated that the *CC* genotype of *rs7257062* was more frequent in the CAD group compared to the control group (10.9% vs 7.7%), with a statistically significant difference (*p* = 0.009). Moreover, the *TT* and *CC + CT* genotype groups of *rs7257062* in the CAD subgroup showed a significant difference in terms of serum triglyceride levels (2.326 ± 1.889 vs 2.059 ± 1.447, *p* = 0.019). Analysis of total cholesterol (TC), low-density lipoprotein cholesterol (LDL-C), high-density lipoprotein cholesterol (HDL-C), apolipoprotein A (ApoA), and apolipoprotein B (ApoB) levels revealed no significant differences between the *TT* and *CC + CT* genotypes. Furthermore, no significant differences in serum lipid levels were observed between genotypes of the other SNPs. Multivariable logistic analysis, controlling for gender, age, body mass index (BMI), triglycerides (TG), TC, HDL-C, LDL-C, ApoA and ApoB, demonstrated that *rs7257062* was still an independent risk factor of CAD (OR = 1.499, 95% CI: 1.036–2.168, *p* = 0.032). MDR analysis revealed that the *rs7257062* interacted significantly with environmental factors such as smoking, diabetes, hypertension, BMI, and TG (*p* < 0.05).

**Conclusions:**

The *rs7257062 *variation of the *C3* gene could be linked to both lipid balance and the risk of CAD. It is conceivable that the interplay between *C3* polymorphisms and environmental elements could account for the etiology of CAD.

## 1. Introduction

Coronary artery disease (CAD), caused by atherosclerotic plaque in the coronary arteries, is a global health risk, as it can reduce or completely block the flow of blood in the vascular lumen, leading to myocardial ischemia and hypoxia. Evidence suggests that both genetic and environmental elements may be involved in the development of this condition. Genetic studies have revealed significant information regarding the molecular cause of CAD. It has been observed that the genetic polymorphism of the C3 component of the complement is strongly correlated with CAD. Moreover, a single nucleotide polymorphism (SNP) in this gene may be a risk factor for CAD [1].

Ever since Rudolf Virchow made his groundbreaking discovery in the mid-1800s, numerous investigations have demonstrated that inflammation caused by the immune system plays a major role in the emergence and advancement of CAD [2]. The *C3* gene is situated on the short arm of chromosome 19 at 19p13.3-2, measuring 41kb in length. The C3 protein, when fully developed, contains 1663 amino acids and has a molecular weight of 184 kD [1]. C3 is a type of adipocytokine, which is expressed in adipose tissue of obese patients and is associated with increased circulating levels [3]. The liver is the main source of C3 production, with adipose tissue being the primary secretor. Additionally, activated macrophages can also contribute to its secretion. C3 has been found to possess anti-infection and immune regulation functions, and can be implicated in pathological immune responses [4]. Various triggers can activate the various pathways of complement activation. Studies have explored a multitude of pathways in relation to human cardiovascular health and metabolism, with indications that they could be implicated in the formation of conditions including insulin resistance and diabetes, hypertension, non-alcoholic fatty liver disease, and atherosclerosis [5].

Numerous studies have examined the association between gene polymorphisms and the likelihood of CAD, however, comparatively few have explored the correlation between *C3* polymorphism and CAD risk. Investigating the association between a particular compound and its influence on the body can be most effectively done by measuring the concentration of that compound in the bloodstream. Jiang H *et al*. [6] conducted a case-control study which revealed that serum C3 levels were significantly higher in coronary heart disease patients than in the healthy control group. The results of the study indicated that individuals with higher C3 levels had a greater likelihood of having severe CAD. Results from the CODAM (Cohort on Diabetes and Atherosclerosis Maastricht) study [7] suggest that individuals who smoke heavily and have elevated C3 levels are more likely to develop coronary heart disease. King R and colleagues discovered that by blocking the connection between complement C3 and fibrinogen, there may be a decrease in cardiovascular events for diabetic patients [8]. Széplaki G *et al*. [9] conducted a prospective study with 266 participants who had severe coronary heart disease, and observed them for 5 years. The research revealed that males with the disease had a significantly higher concentration of C3, implying that C3 can potentially be used as a biomarker for the condition. Additionally, the study demonstrated a positive correlation between C3 and triacylglycerol, as well as a negative correlation between C3 and adiponectin. It has been established by a recent study that the C3*F genotype is significantly associated with myocardial infarction in the Tunisian population [10]. A further study revealed that a higher C3 level is linked to the presence and degree of arterial calcification in middle-aged women, and could be a potential non-invasive marker for early diagnosis of atherosclerosis [11]. C3 is a major contributor to the development and progression of coronary artery disease.

This study sought to investigate the potential correlation between *C3* gene polymorphisms and the development of CAD and lipid profiles in a Chinese population living in Xinjiang region. Furthermore, it aimed to analyze the interactions between SNPs and SNPs with environmental factors associated with CAD risk. The association between *C3* tag SNPs and these conditions was examined to gain further insights.

## 2. Materials and Methods

### 2.1 Study Population

**Fig. 1.**
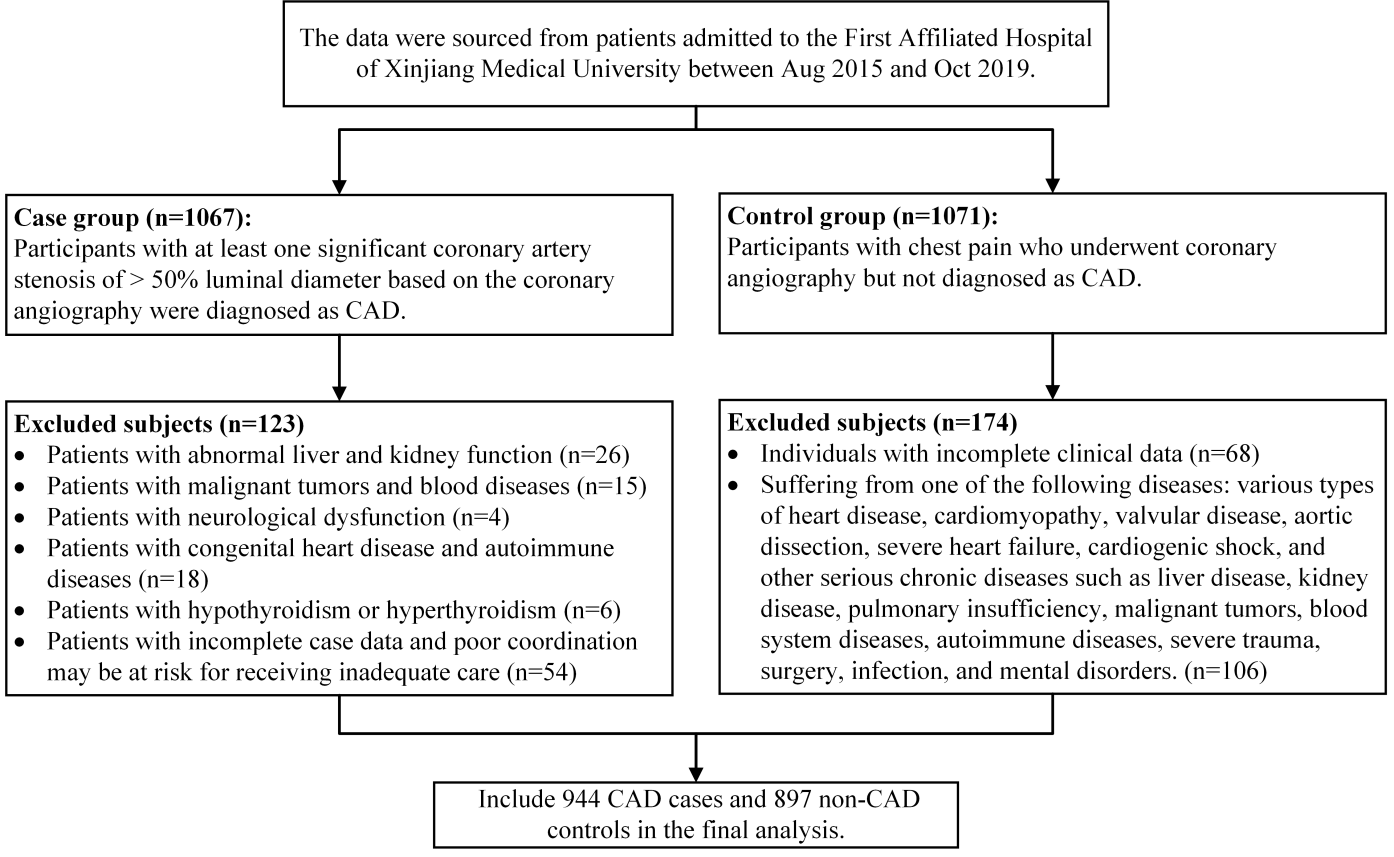
**Inclusion and exclusion criteria used to select the study subjects**. CAD, coronary artery disease.

A total of 1883 unrelated adult subjects were recruited for this study at the First Affiliated Hospital of Xinjiang Medical University between August 2015 and October 2019. The study population included 944 CAD patients (565 males, 288 females; mean age 55.97 ± 10.18 years) and 897 non-CAD controls (583 males, 314 females; mean age 55.94 ± 9.16 years). Fig. 1 shows the inclusion and exclusion criteria used to select the study subjects. The study sample consisted of Chinese patients who had undergone coronary angiography. Patients with CAD were classified as having CAD if they met the diagnostic criteria for the condition: participants with at least one significant coronary artery stenosis (left main, left anterior descending, left circumflex, right coronary artery, and large branches) of >50% luminal diameter based on the coronary angiography [12] were diagnosed as CAD. Exclusion criteria: ① Patients with abnormal liver and kidney function; ② Patients with malignant tumors and blood diseases; ③ Patients with neurological dysfunction; ④ Patients with congenital heart disease and autoimmune diseases; ⑤ Patients with hypothyroidism or hyperthyroidism; ⑥ Patients with incomplete case data and poor coordination may be at risk for receiving inadequate care. Among patients with chest pain who underwent coronary angiography, those without CAD served as controls. Exclusion criteria: ① individuals with incomplete clinical data; ② Suffering from one of the following diseases: various types of heart disease, cardiomyopathy, valvular disease, aortic dissection, severe heart failure, cardiogenic shock, and other serious chronic diseases such as liver disease, kidney disease, pulmonary insufficiency, malignant tumors, blood system diseases, autoimmune diseases, severe trauma, surgery, infection, and mental disorders. All the subjects were non-blood related individuals who had lived in Xinjiang for a long time and signed an informed consent form before being included in the study.

The Ethics Committee of the First Affiliated Hospital of Xinjiang Medical University approved this research project (Approval No. K202309-08).

### 2.2 DNA Extraction and SNP Scan High-Throughput Genotyping

**Fig. 2.**
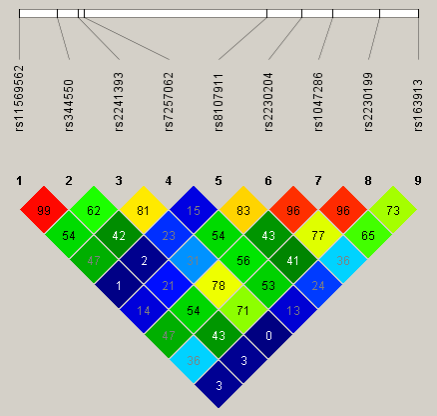
**Linkage disequilibrium (LD) map covering *C3* gene**.

The International HapMap Project website (https://www.ncbi.nlm.nih.gov/snp) and the Haploview 4.2 software (Broad Institute, Cambridge, MA, USA) were used to select 9 tag SNPs of the *C3* gene SNPs-*rs1047286*, *rs11569562*, *rs163913*, *rs2230199*, *rs2230204*, *rs2241393*, *rs7257062*, *rs344550*, *rs8107911* (Table 1) with linkage disequilibrium patterns ($r^{2}$ ≥0.8) and minor allele frequency (MAF ≥0.05). The composition of peripheral venous blood was studied by collecting four milliliters in EDTA tubes (BD Vacutainers, Franklin Lakes, NJ, USA). The AxyPrep DNA Blood kit was used to extract DNA from whole blood. The extracted DNA was then stored at –80 °C for further analyses. All selected SNPs were genotyped using a SNP scan Kit from Genesky Biotechnologies Inc (Cat#: G0104, Shanghai, China). The improved multiplex ligation detection reaction (iMLDR) was used for SNP genotyping without knowledge of any patient clinical data, in a blinded methodology. Approximately 10% of all the genotyped samples were used to monitor quality, with the aim of checking for any discrepancies (Fig. 2).

**Table 1.** **Candidate SNP sites of *C3* gene and related identification primer information**.

SNPs	F-seq (5′-3′)	R-seq (5′-3′)
*rs1047286*	*GCCTCACCTGAGTGCAAGATGA*	*AAGCGCATTCCGGTACCATAGA*
*rs11569562*	*CCATGTCACCATCCACACACAG*	*AGTGAGTGTGAGGTCCTGAAGTTACG*
*rs163913*	*GCTACTGATTACCGCCCTGAGC*	*CAATTCATTTCATGCAGGGCTCA*
*rs2230199*	*GCCAGGGGTGTAGATGGTCTTG*	*GGAACAGACCCCTGACAATGC*
*rs2230204*	*TGGGTCACTGGCCCTTACCTTA*	*TGTCTTTCCACTCTAGCCCAGCA*
*rs2241393*	*GAAGGTGGCCTAGAACCCACAA*	*GCATCCTCAGGGTCGCTAGACA*
*rs7257062_rs344548*	*TGTGACATTGGGAGCCTGGTAG*	*GTGATTGCAGTGGAGTTGAGAATCA*
*rs344550*	*TCCCTGTCTCCAGGTGGCTAAC*	*GGCAGCAGGGTCAACATCAC*
*rs8107911*	*TGCGATTGCCGGTGTGAG*	*TGCCAAACTCGATGAGTGAACAG*

SNP, single nucleotide polymorphism.

### 2.3 Collection of Clinical Data and Detection of Inspection Indicators

Smoking history, drinking history and past concomitant disease history were collected through questionnaire surveys. Height, weight, and biochemical data are obtained from medical health records and measurements conducted in hospital laboratories. Definition of drinking history: frequency of drinking at least once a week. Definition of Hypertension: according to the international Guidelines for the Prevention and Treatment of Hypertension, a diagnosis of hypertension may be made if a patient has their blood pressure checked three times in one day and their systolic blood pressure readings are all ≥140 mmHg and their diastolic blood pressure readings are all ≥90 mmHg; If the patient has a blood pressure reading of less than 140/90 mmHg, but is taking medication for hypertension, they may still be diagnosed with the condition [13]. Definition of diabetes: according to the World Health Organization, diabetes is diagnosed when a person has high blood sugar levels: the concentration of glucose in the blood after an overnight fast is above 7.0 mmol/L (including 7.0); with symptoms of diabetes, the plasma glucose level of the patient measured at any time is higher than 11.1 mmol/L (including 11.1); the blood glucose level within 2 hours after oral glucose tolerance test was found to be increased, exceeding 11.1 mmol/L (including 11.1); the patient was diagnosed with diabetes more than one year after being examined in the hospital [14]. The First Affiliated Hospital of Xinjiang Medical University Laboratory provided test indicators to detect serum total cholesterol (TC), triglyceride (TG), high-density lipoprotein cholesterol (HDL-C), low-density lipoprotein cholesterol (LDL-C), apolipoprotein A1 (ApoA1), apolipoprotein B (ApoB), and other biochemical indicators. All participants were asked to fast for 12 hours prior to providing a 5 mL blood sample. 2 mL of this blood was used to measure serum lipid levels, and the remaining 3 mL was stored in tubes containing anticoagulants (4.80 g/L citric acid, 14.70 g/L glucose, and 13.20 g/L tri-sodium citrate) for DNA extraction. The levels of TC, TG, HDL-C, and LDL-C in the serum were measured by enzymatic methods with commercially available kits (RANDOX Laboratories). The levels of ApoA1 and ApoB in the serum were detected by an immunoturbidimetric immunoassay. The normal values for TC, TG, HDL-C, LDL-C, ApoA1, and ApoB in our Clinical Science Experiment Center were within the following ranges: 3.10–5.17 mmol/L for TC, 0.56–1.70 mmol/L for TG, 0.91–1.81 mmol/L for HDL-C, 2.70–3.20 mmol/L for LDL-C, 1.00–1.78 g/L for ApoA1, and 0.63–1.14 g/L for ApoB.

### 2.4 Statistical Analysis

The data was analyzed using SPSS version 25.0 for Windows (IBM Corp., Armonk, NY, USA). The presence of coronary heart disease (CHD) risk alleles was coded as 0, 1, or 2, and the genotype distribution was assessed using a Chi-square test. The Hardy-Weinberg Equilibrium (HWE) test was used to examine the differences in genotype and allele frequencies between different groups. The Student’s *t*-test or analysis of variance was used to compare clinical parameters between cases and controls. Qualitative variables were reported as frequencies and percentages and evaluated using the Chi-square test. Multivariate logistic regression analyses were performed for SNPs and other risk factors associated with CHD. The odds ratio (OR) and 95% confidence interval (CI) were then calculated in order to evaluate the contribution of the major risk factors. Finally, we used multifactor dimensionality reduction (MDR) to explore potential interactions among SNPs, SNPs and environmental risk factors in CAD.

## 3. Results

### 3.1 The Essential Features of the Subjects

This study included 959 CAD patients and 924 healthy controls. The controls were matched for age and sex. As shown in Table 2, the levels of ApoA (1.08 vs 1.09, *p *= 0.699), BMI (25.81 vs 26.09, *p* = 0.346), and drinking history (30.3% vs 29.5%, *p *= 0.379) were not significantly different between the CAD group and the healthy controls. There were significant differences between the CAD group and the control group in terms of smoking history (45.6% vs 35.9%, *p* < 0.001), diabetes (18.7% vs 15.7%, *p* < 0.001), hypertension (54.2% vs 45.8%, *p* < 0.001), TG (1.90 vs 1.72, *p* = 0.012), TC (4.12 vs 3.82, *p* = 0.001), HLD-C (0.91 vs 1.00, *p* < 0.003), LDL-C (2.61 vs 2.41, *p* < 0.001), and ApoB (0.89 vs 0.84, *p* < 0.001).

**Table 2.** **Clinical characteristics of the subjects**.

Characteristics	CAD	Control	*t/χ^2^*	*p*
Age/year	55.97 ± 10.182	55.94 ± 9.162	–0.075	0.940
Male/Female	656 (69.5%)/288 (30.5%)	583 (65.0%)/314 (35.0%)	4.227	0.042
Smoking history (%)	430 (45.6%)	322 (35.9%)	18.072	<0.001
Drinking history (%)	290 (30.8%)	262 (29.2%)	0.544	0.476
Diabetes (%)	174 (18.7%)	136 (15.7%)	3.580	<0.001
Hypertension (%)	473 (54.2%)	400 (45.8%)	6.886	0.005
BMI (${\mathrm{kg/m}}^{2}$)	25.91 ± 3.343	26.09 ± 3.719	0.779	0.346
TG (mmol/L)	1.90 ± 1.679	1.72 ± 1.346	–2.522	0.012
TC (mmol/L)	4.12 ± 1.727	3.82 ± 1.433	–4.010	<0.001
HDL-C (mmol/L)	0.91 ± 0.383	1.00 ± 0.440	4.909	0.003
LDL-C (mmol/L)	2.61 ± 1.231	2.41 ± 1.037	–3.822	<0.001
ApoA (mmol/L)	1.08 ± 0.452	1.09 ± 0.409	0.387	0.699
ApoB (mmol/L)	0.89 ± 0.300	0.84 ± 0.255	–3.561	<0.001

CAD, coronary artery disease; BMI, body mass index; TC, total cholesterol; TG, triglyceride; HDL-C, high density lipoprotein cholesterol; LDL-C, low density lipoprotein cholesterol; ApoA, apolipoprotein A; ApoB, apolipoprotein B.

### 3.2 Univariate Analysis of the Genotype and Allele Distributions of C3 Related SNPs

The genotypic and allelic distributions of *rs1047286*, *rs11569562*, *rs163913*, *rs2230199*, *rs2230204*, *rs2241393*, *rs7257062*, *rs344550 *and *rs8107911* were exhibited in Table 3. The distributions of *CC* (10.9% vs 7.7%), *CT* (44.2% vs 43.0%), and *TT* (44.9% vs 49.3%) genotypes in *rs7257062* of the CAD patients were statistically different relative to the control group. Compared with the *T* allele (67.0% vs 70.8%), the *C* allele frequency showed an increased level in the CAD group relative to the control group (33.0% vs 29.2%), *p* < 0.05. No significant differences were observed in genotype or allele frequencies of the remaining tag SNPs between CAD patients and controls. The deviation of the nine SNPs was analyzed using the Hardy-Weinberg equilibrium. As shown in Table 3, the genotype frequencies of the four SNPs did not deviate from the equilibrium (all *p* > 0.05).

**Table 3.** **Genotypic and allelic distribution frequencies of the SNPs in controls and CAD**.

SNP/Genotype		Control	CAD	*p*	OR (95% CI)
*rs1047286*					
	*AA*	3 (0.3%)	3 (0.3%)	0.951	1.051 (0.212–5.223)
	*GA*	57 (6.4%)	60 (6.4%)	0.999	1 (0.687–1.454)
	*GG*	837 (93.3%)	881 (93.3%)		Reference
	*A*	63 (1.7%)	66 (1.8%)	0.979	1.005 (0.707–1.428)
	*G*	1731 (47.0%)	1822 (49.5%)		
*rs11569562*					
	*AA*	203 (22.7%)	221 (23.4%)	0.701	0.958 (0.771–1.191)
	*GA*	462 (51.6%)	494 (52.3%)	0.742	0.97 (0.808–1.164
	*GG*	231 (25.8%)	229 (24.3%)		Reference
	*A*	868 (48.4%)	936 (49.6%)	0.490	0.955 (0.84–1.087)
	*G*	924 (51.6%)	952 (50.4%)		
*rs163913*					
	*CC*	93 (10.5%)	105 (11.2%)	0.609	0.926 (0.689–1.244)
	*TC*	415 (46.8%)	440 (47.1%)	0.907	0.989 (0.823–1.189)
	*TT*	379 (42.7%)	390 (41.7%)		Reference
	*C*	601 (33.9%)	650 (34.8%)	0.576	0.962 (0.839–1.103)
	*T*	1173 (66.1%)	1220 (65.2%)		
*rs2230199*					
	*CC*	4 (0.4%)	2 (0.2%)	0.378	2.11 (0.385–11.547)
	*GC*	63 (7.0%)	76 (8.1%)	0.404	0.863 (0.61–1.221)
	*GG*	830 (92.5)	866 (91.7%)		Reference
	*C*	71 (4.0%)	80 (4.2%)	0.669	0.931 (0.672–1.291)
	*G*	1723 (96.0%)	1808 (95.8%)		
*rs2230204*					
	*CC*	265 (29.5%)	309 (32.7%)	0.140	0.862 (0.707–1.050)
	*TC*	461 (51.4%)	454 (48.1%)	0.157	1.141 (0.95–1.37)
	*TT*	171 (19.1%)	181 (19.2%)		Reference
	*C*	991 (48.0%)	1072 (52.0%)	0.347	0.939 (0.825–1.07)
	*T*	803 (49.6%)	816 (50.4%)		
*rs2241393*					
	*CC*	308 (34.3%)	312 (33.1%)	0.560	1.059 (0.873–1.285)
	*GC*	434 (48.4%)	480 (50.8%)	0.291	0.906 (0.755–1.088)
	*GG*	155 (17.3%)	152 (16.1%)		Reference
	*C*	1050 (58.5%)	1104 (58.5%)	0.974	1.002 (0.879–1.143)
	*G*	744 (41.5%)	784 (41.5%)		
*rs344550*					
	*CC*	120 (13.4%)	115 (12.2%)	0.442	1.113 (0.847–1.464)
	*GC*	429 (47.8%)	460 (48.7%)	0.698	0.964 (0.803–1.158)
	*GG*	348 (38.8%)	369 (39.1%)		Reference
	*C*	669 (37.3%)	690 (36.5%)	0.640	1.032 (0.903–1.18)
	*G*	1125 (62.7%)	1198 (63.5%)		
*rs7257062*					
	*CC*	69 (7.7%)	103 (10.9%)	**0.009	1.556 (1.116–2.170)
	*CT*	385 (43.0%)	417 (44.2%)	0.216	1.129 (0.932–1.368)
	*TT*	442 (49.3%)	424 (44.9%)		Reference
	*C*	523 (29.2%)	623 (33.0%)	*0.012	1.197 (1.041–1.377)
	*T*	1271 (70.8%)	1265 (67.0%)		
*rs8107911*					
	*AA*	691 (77.0%)	735 (77.9%)	0.672	0.954 (0.766–1.187)
	*GA*	193 (21.5%)	197 (20.9%)	0.734	1.04 (0.831–1.3)
	*GG*	13 (1.4%)	12 (1.3%)		Reference
	*A*	1757 (88.9%)	1667 (88.3%)	0.543	1.064 (0.872–1.297)
	*G*	219 (11.1%)	221 (11.7%)		

SNP, single nucleotide polymorphism; CAD, coronary artery disease; OR, odds ratio; CI, confidence interval. *: *p* < 0.05; **: *p* < 0.01.

### 3.3 Logistic Analysis of the C3 Related SNPs in CAD

The results of the multivariable logistic regression analysis are shown in Table 4. We included variables with significant differences identified in Table 2, as well as the *rs7257062 CC* genotype, in the multivariable logistic regression. After adjusting the confounders including gender, smoking history, diabetes, hypertension, TG, TC, HDL-C, LDL-C and ApoB, the *CC* genotype of *rs7257062 *was identified to be an independent risk factor for CAD (OR = 1.581, 95% CI: 1.094–2.284, *p* = 0.015). The power of our study on *CC* genotype of *rs7257062* is 76.29%. Our study was adequately powered to detect this association.

**Table 4.** **Multivariate logistic regression analysis results**.

Variables	B	S.E.	Wald	OR	95% CI	*p* value
Male/Female	0.031	0.140	0.049	1.031	0.784–1.356	0.825
Smoking history (%)	0.333	0.129	6.668	1.395	1.084–1.796	0.010*
Diabetes (%)	0.260	0.138	3.566	1.297	0.990–1.699	0.059
Hypertension (%)	0.303	0.107	8.111	1.354	1.099–1.669	0.004*
TG (mmol/L)	–0.061	0.045	1.878	0.940	0.861–1.027	0.171
TC (mmol/L)	0.463	0.112	17.137	1.589	1.276–1.979	<0.001*
HDL-C (mmol/L)	–1.566	0.225	48.246	0.209	0.134–0.325	<0.001*
LDL-C (mmol/L)	–0.051	0.132	0.148	0.951	0.734–1.231	0.700
ApoB (mmol/L)	–0.329	0.255	1.660	0.720	0.437–1.187	0.198
*rs7257062 CC* genotype	0.458	0.188	5.949	1.581	1.094–2.284	0.015*

*: *p* < 0.05. S.E., standard error; OR, odds ratio; CI, confidence interval; HDL-C, high density lipoprotein cholesterol; LDL-C, low density lipoprotein cholesterol; ApoB, apolipoprotein B; TC, total cholesterol; TG, triglyceride; B, regression coefficient.

### 3.4 The Association of C3 Genotypes and the Levels of Lipid Parameters in CAD

Table 5 demonstrates that there are significant differences in the serum levels of TG between the genotypes of *rs7257062*. Since the *C* allele was major allele, we grouped the *CC + CT* genotype as carriers of the *C* allele. The mean values for the *TT *and *CC + CT* genotypes in the CAD subgroup are significantly different (2.326 ± 1.889 vs 2.059 ± 1.447, *p* = 0.019). The levels of TC, LDL-C, HDL-C, ApoA, and ApoB did not differ significantly between the *TT* and *CC + CT* genotypes. No significant difference in serum levels was observed between alleles of the remaining SNPs.

**Table 5.** **Effect of nine dominant genotypes on serum lipid levels in CAD and control groups**.

SNP	Genotypes	TG	TC	HDL-C	LDL-C	ApoA	ApoB
*rs1047286*	CAD subgroup						
	*GG*	2.749 ± 0.158	4.471 ± 0.045	0.984 ± 0.010	2.841 ± 0.035	1.179 ± 0.012	0.906 ± 0.012
	*AA + GA*	2.985 ± 0.311	4.342 ± 0.182	0.965 ± 0.034	2.712 ± 0.136	1.168 ± 0.032	0.846 ± 0.030
	*p*	0.389	0.472	0.608	0.361	0.755	0.069
	Control group						
	*GG*	1.89 ± 0.049	4.15 ± 0.035	1.07 ± 0.010	2.63 ± 0.029	1.19 ± 0.009	0.85 ± 0.009
	*AA + GA*	1.75 ± 0.155	4.03 ± 0.120	1.12 ± 0.055	2.54 ± 0.111	1.22 ± 0.043	0.83 ± 0.034
	*p*	0.287	0.189	0.626	0.306	0.588	0.482
*rs11569562*	CAD subgroup						
	*GG*	2.474 ± 0.956	4.392 ± 0.088	0.999 ± 0.018	2.864 ± 0.072	1.172 ± 0.018	0.904 ± 0.022
	*AA + GA*	2.271 ± 0.869	4.487 ± 0.051	0.977 ± 0.011	2.823 ± 0.039	1.18 ± 0.014	0.901 ± 0.014
	*p*	0.065	0.351	0.295	0.610	0.727	0.892
	Control group						
	*GG*	1.96 ± 0.110	4.068 ± 0.067	1.067 ± 0.020	2.525 ± 0.052	1.198 ± 0.019	0.847 ± 0.019
	*AA + GA*	1.856 ± 0.051	4.168 ± 0.038	1.077 ± 0.012	2.659 ± 0.032	1.192 ± 0.010	0.852 ± 0.010
	*p*	0.484	0.418	0.991	0.167	0.767	0.824
*rs163913*	CAD subgroup						
	*TT*	2.272 ± 0.096	4.540 ± 0.070	0.995 ± 0.015	2.872 ± 0.056	1.185 ± 0.014	0.922 ± 0.022
	*CC + TC*	2.159 ± 0.073	4.419 ± 0.057	0.975 ± 0.012	2.811 ± 0.043	1.174 ± 0.017	0.890 ± 0.013
	*p*	0.309	0.180	0.280	0.391	0.636	0.203
	Control group						
	*TT*	1.837 ± 0.067	4.132 ± 0.050	1.069 ± 0.016	2.618 ± 0.042	1.189 ± 0.014	0.850 ± 0.014
	*CC + TC*	1.916 ± 0.066	4.154 ± 0.045	1.081 ± 0.014	2.632 ± 0.038	1.199 ± 0.012	0.852 ± 0.012
	*p*	0.314	0.700	0.577	0.786	0.598	0.937
*rs2230199*	CAD subgroup						
	*GG*	2.033 ± 0.117	4.475 ± 0.046	0.983 ± 0.010	2.841 ± 0.036	1.178 ± 0.012	0.905 ± 0.013
	*CC + GC*	2.188 ± 0.085	4.324 ± 0.153	0.976 ± 0.036	2.737 ± 0.121	1.179 ± 0.030	0.863 ± 0.030
	*p*	0.207	0.349	0.854	0.410	0.971	0.190
	Control group						
	*GG*	1.891 ± 0.050	4.152 ± 0.035	1.071 ± 0.010	2.633 ± 0.289	1.194 ± 0.009	0.853 ± 0.010
	*CC + GC*	1.774 ± 0.146	4.015 ± 0.114	1.111 ± 0.050	2.523 ± 0.106	1.191 ± 0.041	0.821 ± 0.032
	*p*	0.337	0.132	0.73	0.225	0.947	0.357
*rs2230204*	CAD subgroup						
	*TT*	2.069 ± 0.135	4.643 ± 0.104	0.995 ± 0.020	2.966 ± 0.084	1.197 ± 0.023	0.933 ± 0.022
	*CC + TC*	2.234 ± 0.064	4.420 ± 0.048	0.979 ± 0.010	2.800 ± 0.037	1.173 ± 0.013	0.894 ± 0.014
	*p*	0.259	0.053	0.485	0.074	0.364	0.137
	Control group						
	*TT*	1.896 ± 0.104	4.052 ± 0.079	1.051 ± 0.021	2.598 ± 0.064	1.188 ± 0.020	0.865 ± 0.023
	*CC + TC*	1.879 ± 0.053	4.164 ± 0.037	1.080 ± 0.012	2.631 ± 0.031	1.195 ± 0.010	0.847 ± 0.001
	*p*	0.893	0.175	0.186	0.567	0.755	0.466
*rs2241393*	CAD subgroup						
	*GG*	2.347 ± 0.105	4.484 ± 0.115	0.999 ± 0.256	2.761 ± 0.084	1.211 ± 0.048	0.872 ± 0.026
	*CC + GC*	2.163 ± 0.080	4.460 ± 0.048	0.979 ± 0.010	2.846 ± 0.037	1.172 ± 0.010	0.927 ± 0.013
	*p*	0.194	0.845	0.472	0.356	0.441	0.231
	Control group						
	*GG*	1.876 ± 0.109	4.122 ± 0.075	1.065 ± 0.026	2.598 ± 0.064	1.173 ± 0.022	0.864 ± 0.020
	*CC + GC*	1.884 ± 0.052	4.146 ± 0.037	1.076 ± 0.011	2.630 ± 0.031	1.197 ± 0.010	0.848 ± 0.010
	*p*	0.612	0.89	0.871	0.937	0.314	0.478
*rs344550*	CAD subgroup						
	*GG*	2.174 ± 0.065	4.469 ± 0.069	1.004 ± 0.015	2.842 ± 0.054	1.182 ± 0.014	0.907 ± 0.016
	*CC + GC*	2.307 ± 0.127	4.460 ± 0.057	0.968 ± 0.012	2.827 ± 0.044	1.176 ± 0.017	0.899 ± 0.016
	*p*	0.576	0.920	0.056	0.824	0.790	0.723
	Control group						
	*GG*	1.992 ± 0.090	4.109 ± 0.054	1.085 ± 0.018	2.563 ± 0.043	1.204 ± 0.015	0.853 ± 0.015
	*CC + GC*	1.815 ± 0.052	4.162 ± 0.042	1.068 ± 0.013	2.663 ± 0.365	1.187 ± 0.011	0.849 ± 0.011
	*p*	0.078	0.709	0.241	0.209	0.375	0.817
*rs7257062*	CAD subgroup						
	*TT*	2.059 ± 1.447	4.473 ± 1.329	0.976 ± 0.262	2.872 ± 1.080	1.174 ± 0.267	0.906 ± 0.318
	*CC + TC*	2.326 ± 1.889	4.454 ± 1.259	0.987 ± 0.278	2.800 ± 0.937	1.180 ± 0.385	0.898 ± 0.371
	*p*	0.019*	0.833	0.566	0.304	0.779	0.749
	Control group						
	*TT*	1.913 ± 0.070	4.128 ± 0.051	1.066 ± 0.015	2.615 ± 0.041	1.187 ± 0.013	0.851 ± 0.014
	*CC + TC*	1.851 ± 0.062	4.156 ± 0.043	1.083 ± 0.015	2.635 ± 0.038	1.200 ± 0.012	0.850 ± 0.012
	*p*	0.382	0.584	0.395	0.590	0.478	0.951
*rs8107911*	CAD subgroup						
	*GG*	2.456 ± 0.502	4.226 ± 0.263	0.918 ± 0.050	2.649 ± 0.234	1.106 ± 0.054	0.836 ± 0.059
	*AA + GA*	2.199 ± 0.058	4.466 ± 0.044	0.983 ± 0.009	2.835 ± 0.035	1.179 ± 0.012	0.903 ± 0.012
	*p*	0.628	0.389	0.226	0.449	0.213	0.294
	Control group						
	*GG*	1.668 ± 0.375	4.129 ± 0.168	2.574 ± 0.176	1.294 ± 0.076	0.803 ± 0.064	0.803 ± 0.064
	*AA + GA*	1.886 ± 0.048	4.142 ± 0.034	1.071 ± 0.010	2.626 ± 0.028	1.192 ± 0.009	0.851 ± 0.009
	*p*	0.611	0.901	0.065	0.745	0.209	0.466

*: *p* < 0.05. SNP, single nucleotide polymorphism; CAD, coronary artery disease; HDL-C, high-density lipoprotein-cholesterol; LDL-C, low-density lipoprotein-cholesterol; TC, total cholesterol; TG, triglyceride; ApoA, apolipoprotein A; ApoB, apolipoprotein B.

### 3.5 Single Factor Analysis of Gensini Score and Vascular Stenosis Index in Different Genotypes

Gensini score and vascular stenosis number were compared between the genotypes of all nine tagSNPs: *rs1047286*, *rs11569562*, *rs163913*, *rs2230199*, *rs2230204*, *rs2241393*, *rs344550*, *rs7257062 *and *rs8107911*. The results showed that there were no significant differences in any aspect between the two groups (*p* > 0.05) in Table 6.

**Table 6.** **Comparison of Gensini score and vascular stenosis number among the genotypes of all nine tagSNPs**.

SNPs	Genotypes	Gensini score	Number of stenosed vessels
*rs1047286*	*AA*	94.333 ± 10.398	2.67 ± 0.333
	*GA*	52.042 ± 4.485	2.25 ± 0.190
	*GG*	56.214 ± 1.644	2.48 ± 0.052
	F-test	1.174	0.626
	*p*	0.310	0.353
*rs11569562*	*AA*	55.336 ± 2.759	2.36 ± 0.099
	*GA*	55.874 ± 1.766	2.52 ± 0.069
	*GG*	57.198 ± 4.471	2.44 ± 0.104
	F-test	0.071	0.842
	*p*	0.932	0.431
*rs163913*	*CC*	57.177 ± 4.296	1.56 ± 0.338
	*TC*	56.811 ± 2.666	2.61 ± 0.152
	*TT*	54.788 ± 1.938	2.39 ± 0.071
	F-test	0.255	1.353
	*p*	0.775	0.259
*rs2230199*	*CC*	98.500 ± 16.500	2.50 ± 0.500
	*GC*	50.954 ± 4.314	2.25 ± 0.171
	*GG*	56.422 ± 1.658	2.48 ± 0.052
	F-test	1.237	0.778
	*p*	0.291	0.460
*rs2230204*	*CC*	56.912 ± 2.295	2.47 ± 0.089
	*TC*	53.847 ± 1.861	2.48 ± 0.072
	*TT*	60.144 ± 5.382	2.41 ± 0.112
	F-test	1.187	0.100
	*p*	0.306	0.905
*rs2241393*	*CC*	57.672 ± 3.543	2.44 ± 0.083
	*GC*	54.923 ± 1.781	2.45 ± 0.073
	*GG*	56.432 ± 3.178	2.54 ± 0.122
	F-test	0.269	0.264
	*p*	0.764	0.768
*rs344550*	*CC*	59.724 ± 3.803	2.52 ± 0.138
	*GC*	56.124 ± 1.867	2.47 ± 0.072
	*GG*	54.865 ± 3.024	2.44 ± 0.080
	F-test	0.476	0.107
	*p*	0.621	0.898
*rs7257062*	*CC*	49.692 ± 3.391	2.38 ± 0.148
	*TC*	55.537 ± 1.911	2.47 ± 0.078
	*TT*	58.165 ± 2.813	2.47 ± 0.072
	F-test	1.284	0.167
	*p*	0.277	0.846
*rs8107911*	*AA*	57.454 ± 1.862	2.47 ± 0.057
	*GA*	49.917 ± 2.633	2.42 ± 0.112
	*GG*	71.667 ± 12.264	2.58 ± 0.193
	F-test	2.690	0.173
	*p*	0.068	0.841

SNP, single nucleotide polymorphism.

### 3.6 The Interactions between SNPs of C3, SNPs and Environmental Risk Factors of CAD

After applying the ReliefF filter, *rs7257062*, smoking, diabetes, hypertension, BMI, and TG were included in the MDR analysis of SNP–environment interactions. As is shown in Table 7, the six factor model (*rs7257062*, smoking, diabetes, hypertension, BMI, and TG) was determined to be the best model, as it had the highest cross-validation consistency (CVC) of 10/10 with a testing accuracy of 61.59% (0.0457). However, no significant SNP–SNP interaction model was identified.

**Table 7.** **Best multiple-factor interaction models identified by MDR**.

Model		Training accuracy (%)	Testing accuracy (%)	CVC	*p* value
SNPs					
	*rs11569562, rs163913*	55.99	51.23	7/10	0.8304
	*rs2230204, rs2241393, rs8107911*	58.93	48.80	5/10	0.8360
	*rs11569562, rs163913, rs7257062, rs8107911*	62.60	50.66	5/10	0.9091
	*rs11569562, rs163913, rs2230204, rs2241393, rs8107911*	66.84	49.02	5/10	0.8652
	*rs11569562, rs163913, rs2230204, rs2241393, rs7257062, rs8107911*	71.25	49.67	9/10	0.9543
	*rs11569562, rs163913, rs2230204, rs2241393, rs344550, rs7257062, rs8107911*	74.17	48.74	10/10	0.8259
	*rs11569562, rs163913, rs2230199, rs2230204, rs2241393, rs344550, rs7257062, rs8107911*	75.70	47.14	10/10	0.6168
	*rs1047286, rs11569562, rs163913, rs2230199, rs2230204, rs2241393, rs344550, rs7257062, rs8107911*	75.81	47.04	10/10	0.6046
SNPs and environmental factors					
	smoke	60.48	60.48	10/10	0.0691
	*rs7257062*, smoke	61.74	61.74	10/10	0.0431
	*rs7257062*, smoke, TG	63.65	63.15	10/10	0.0235
	*rs7257062*, smoke, BMI, TG	65.37	57.69	4/10	0.1851
	*rs7257062*, smoke, diabetes, BMI, TG	68.49	57.45	6/10	0.1990
	*rs7257062*, smoke, diabetes, hypertension, BMI, TG	71.98	61.59	10/10	0.0457

MDR, multifactor dimensionality reduction; SNP, single nucleotide polymorphism; TG, triglyceride; BMI, body mass index; CVC, cross-validation consistency.

### 3.7 Hierarchical Interaction Graph

**Fig. 3.**
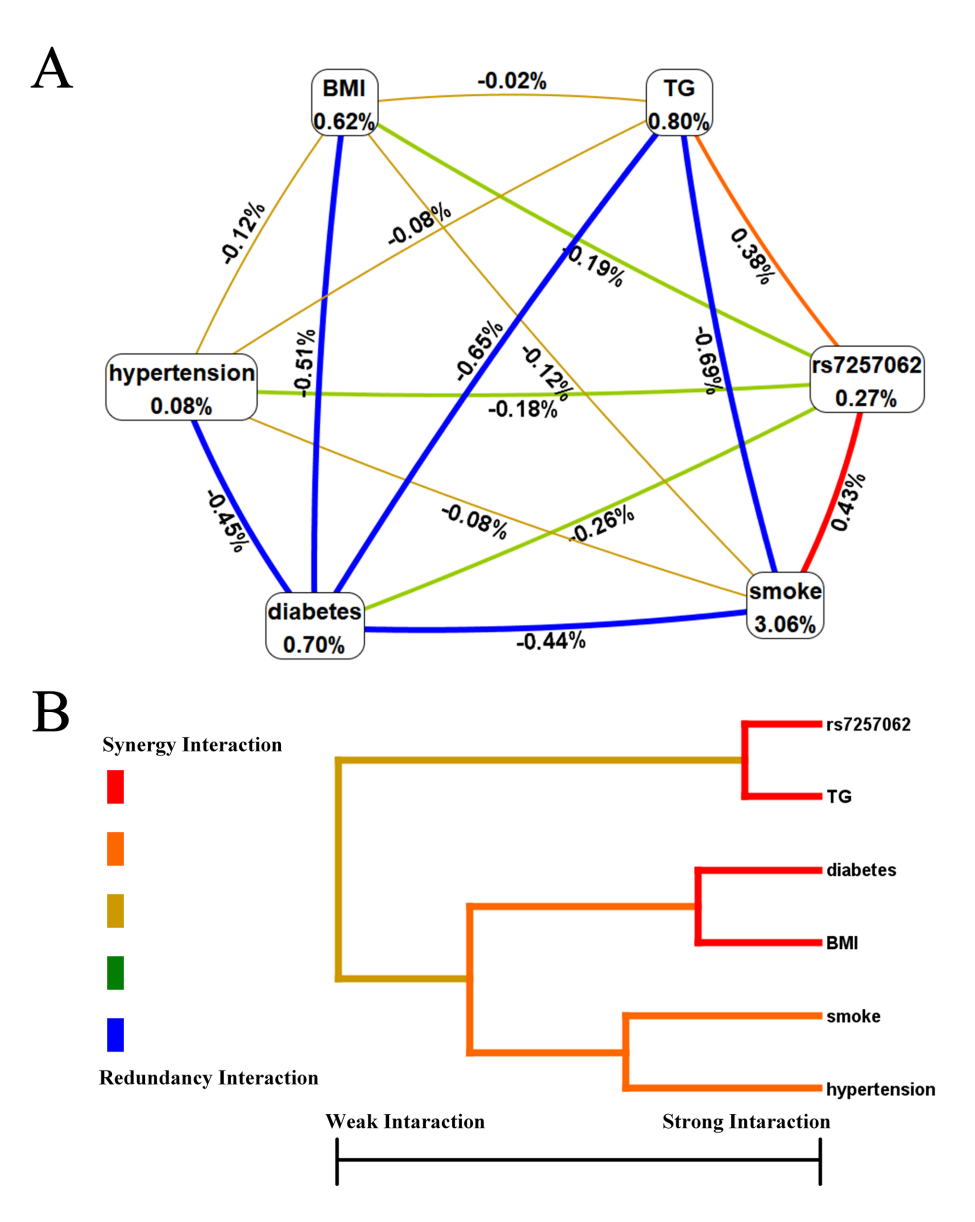
**Hierarchical interaction graph and interaction dendrogram**. (A) The percentage at the bottom of each factor in a hierarchical interaction graph represents its entropy, while the percentage on each line indicates the interaction percentage of entropy between two factors. The red line symbolizes synergy redundancy interaction, and the blue line indicates redundancy interaction. (B) interaction dendrogram illustrates the intensity of interaction from left to right, with the red line signifying stronger synergy interaction and the orange line representing weaker synergy interaction. TG, triglyceride; BMI, body mass index.

After utilizing the multifactor dimensionality reduction (MDR) algorithm to identify a high-risk combination of SNPs and environmental factors, we further employed the concept of information gain to interpret their relationship. Subsequently, we created a hierarchical interaction graph to visualize the interactions (as shown in Fig. 3A). The results revealed a positive correlation between *rs7257062 *and smoke, with an interaction entropy of 0.43%, and between *rs7257062 *and TG with an interaction entropy of 0.38%. The remaining relationships were all negative correlations, with the highest interaction entropies occurring between smoke and TG (–0.69%), TG and diabetes (–0.65%), BMI and diabetes (–0.51%), hypertension and diabetes (–0.45%), and smoke and diabetes (–0.44%).

### 3.8 Interaction Dendrogram

The interaction dendrogram in Fig. 3B reveals that *rs7257062 *and TG have the strongest synergy interaction, as indicated by the red line. In contrast, the *rs7257062 *SNPs is located on a different branch than diabetes, BMI, smoke, and hypertension, indicating a weak synergy interaction, as indicated by the orange line. Moreover, the results suggest that diabetes and BMI have a strong synergy interaction.

## 4. Discussion

To ascertain the connection between *C3* gene polymorphisms and CAD in China, a comprehensive, multicenter study with a large sample size is indispensable. We aimed to investigate whether *C3* gene variants are linked to the risk of CAD and lipid levels in the Chinese population.

This study was conducted to evaluate the roles of *C3* related SNPs (*rs1047286*, *rs11569562*, *rs163913*, *rs2230199*, *rs2230204*, *rs2241393*, *rs344550*, *rs7257062*, and *rs8107911*) in patients with CAD. The results depicted that the *CC *genotype in *rs7257062 *was an independent risk factor for CAD after adjusting gender, smoking history, diabetes, hypertension, TG, TC, HDL-C, LDL-C and ApoB. The CAD subgroup showed significant differences in TG levels between the *TT *and *CC + TC* genotypes in *rs7257062*. There were no differences in Gensini score or vascular stenosis that were statistically significant between the groups for any of these SNPs. *Rs7257062*, smoking, diabetes, hypertension, BMI, and TG were all found to have a significant correlation with CAD risk when taken together. According to Barrington R *et al*. [15], the levels of complement C3 and C4 both increase during chronic inflammation, and a high level of C3 has been found to be associated with myocardial infarction. The study by Muscari A *et al*. [16] found that C3 and C-reactive protein (CRP) were significantly elevated in patients with myocardial infarction and cardiovascular disease, compared to a healthy control group. This suggests that C3 is an independent risk factor for myocardial infarction, and that C3 is a more specific marker. According to Phillips CM *et al*. [17] when C3 levels are elevated, it is associated with insulin resistance, abnormal obesity, and low HDL-C. The study of the relationship between *C3* polymorphism and metabolic syndrome in the French population found that the *rs2250656 *polymorphism was related to C3 levels. The study found that carriers of the A allele for *rs1569562 *had significantly higher levels of C3 than patients with the *GG *genotype; the study also found that the *AA *genotype had higher levels of C3 than the *GG *genotype. Nsaiba MJ *et al*. [18] found that the *rs2230199 *polymorphism of *C3* was associated with blood lipids in a study of Tunisian patients with schizophrenia. Their study results showed that patients with the *CG *and *GG *genotypes of *rs2230199 *had significantly higher levels of TG and TC, respectively, compared to those with the *CC *genotype. This suggests that the *GG *genotype of *rs223019 *may be associated with higher levels of HDL-C. The study conducted by Torres T *et al*. [19] revealed that C3 levels were linked with various conditions like abdominal visceral fat, insulin resistance, and the metabolic syndrome. These conditions are often attributed to oxidized LDL-C in patients with psoriasis. Garcia-Arguinzonis M *et al*. [20] indicated in their research that C3 complement pathway could be a novel player in vascular remodeling and in the progression of advanced human atherosclerotic lesions. Copenhaver MM* et al*.’s [21] study found that C3 plasma concentrations increased in obese individuals, which may contribute to the early onset of cardiovascular disease. The findings of a study conducted by Dissing J *et al*. [22] indicate that the *C3* gene polymorphism may play a role in the development of atherosclerotic disease in older individuals residing in Copenhagen. According to the research of Leban *et al*. [10], individuals with the C3*F genotype had a greater chance of suffering from a myocardial infarction. They estimated that this polymorphism significantly increased the likelihood of myocardial infarction.

It has been proven that the elevated levels of TG, TC, LDL-C, ApoB and Lp(a), together with the decreased HDL-C and ApoA1 levels, are indicators of dyslipidemia, which is associated with the pathogenesis of CAD [23]. The alternative C3 complement system, as a key inflammatory mediator, seems to be involved in the atherosclerotic process, studies have reported an increase in the expression of complement cascade components, including C3-derived products, in Familial hypercholesterolemia patients who show no clinical signs of coronary artery disease [24]. We found that, compared to the control group, patients with CAD had noticeably higher levels of TC, TG, ApoB, and LDL-C. There was a significant difference in the level of HDL-C between the control group and the CAD group, with the control group having a higher level. ApoA levels were similar between these two groups.

The relationship between *C3* polymorphisms and susceptibility to CAD is not well-established in the literature. We selected nine SNPs as tagSNPs for our investigation. No association was found between *rs1047286*, *rs11569562*, *rs163913*, *rs2230199*, *rs2230204*, *rs2241393*, *rs344550*, *rs8107911 *polymorphisms and CAD or with lipid levels, except for *rs7257062*. *rs7257062 *was found to be an independent risk factor of CAD (OR = 1.581, 95% CI: 1.094–2.284, *p* = 0.015) in a multivariable logistic analysis, after controlling for potential confounders including gender, smoking history, diabetes, hypertension, TG, TC, HDL-C, LDL-C and ApoB. The distribution of the *CC *genotype of *rs7257062 *between the CAD and control groups were 103 (10.9%) and 69 (7.7%), *p* = 0.009. Individuals with the *C *allele in *rs7257062 *genotype had higher TG levels than in the *TT *and *CT *genotype in the control and CAD groups (*TT *= 2.059 ± 1.447, *CC* + *CT* 2.326 ± 1.889, *p* = 0.019). Similar results were found by Chen Y *et al*. [25], their findings suggest that individuals with the *C* allele of the rs*7257062* (*301T >C*) polymorphism had higher levels of TG and TC, which suggests that this genetic variation may be associated with an increased risk of CAD in Uygur and Han populations in China. Our MDR analysis revealed a possible correlation between *rs7257062*, smoke, diabetes, hypertension, BMI, and TG. Similarly to CAD, diabetes and hypertension are complex disorders that involve multiple genes and factors [26]. Results from a study of 75 non-Hispanic white adolescents showed that complement components C3 and their genetics are linked to cardiometabolic risk [21]. These findings identified the interaction between *C3* and environmental risk factors could contribute to a better understanding of genetic susceptibility to CAD. However, we did not find an increase in total cholesterol levels between the different genotypes of the *rs7257062 *(*301T>C*) gene in the CAD group. The findings of Cai G *et al*. [27] also suggest that the *C3* polymorphism may be associated with lipid levels. However, the relationship between *C3* and the severity of CAD is a source of contention among researchers. Some suggest that there is a correlation between the two, while others refute this claim. A case-control study by Jiang H *et al*. [6] found that the serum C3 level of moderate and severe coronary heart disease subgroups was higher than mild coronary heart disease subgroup. On the other hand, in the ATHEROREMO-IVUS study, Battes LC *et al*. [28] found that the level of complement C3 was not significantly related to cardiovascular events or coronary plaque stability. A study of 338 patients with severe coronary heart disease and 490 healthy controls in Budapest, Hungary by Császár A [29], found that the C3*F genotype was significantly more common in the former group. This suggests that patients with coronary heart disease who have the *C3F* allele are at an increased risk for developing myocardial infarction. Leban N *et al*. [10] also found that the prevalence of myocardial infarction of *C3* allele carriers increased significantly. In a study of 400 individuals, Golabi P *et al*. [30] found that the genotype and allele frequencies of *C3* did not differ significantly between patients with myocardial infarction and controls.

In our study, we found no differences between the alleles of nine SNPs and the Gensini score and vascular stenosis numbers in terms of the relationship between the *C3* polymorphisms and the severity of CAD.

## 5. Conclusions

The aim of this study was to explore the correlation between *C3* related tag SNPs and CAD. Our findings showed that CC genotype in *rs7257062 *was an independent risk factor for CAD. We also discovered a noteworthy correlation between *rs7257062*, smoke, diabetes, hypertension, BMI, and TG, which is affected by the environment. The *rs7257062 *variant impacted the levels of TG in CAD patients. *C3* gene polymorphisms were associated with both lipid metabolism and CAD susceptibility in the Chinese population in Xinjiang region. Our outcome may indicate a potential route of investigation into the origin of Coronary Artery Disease.

The present study had several limitations that should be acknowledged. Firstly, the sample size was relatively small and limited to a single region, which might have reduced the statistical efficacy of our findings. Secondly, the biological function of *C3* gene was not validated in this work. Larger studies with functional assays are needed to confirm the conclusions of this study.

## Data Availability

Our research data is not available for sharing due to the sensitive nature of the data. The data used in this study contain personally identifiable information and are subject to privacy regulations and ethical considerations. As a result, we are unable to share the data with external parties to ensure the protection of participants’ confidentiality and privacy.

## References

[1] Delanghe JR, Speeckaert R, Speeckaert MM (2014). Complement C3 and its polymorphism: biological and clinical consequences. *Pathology*.

[2] Moriya J (2019). Critical roles of inflammation in atherosclerosis. *Journal of Cardiology*.

[3] Copenhaver M, Yu CY, Hoffman RP (2019). Complement Components, C3 and C4, and the Metabolic Syndrome. *Current Diabetes Reviews*.

[4] Hertle E, van Greevenbroek MMJ, Stehouwer CDA (2012). Complement C3: an emerging risk factor in cardiometabolic disease. *Diabetologia*.

[5] Ursini F, Abenavoli L (2018). The Emerging Role of Complement C3 as A Biomarker of Insulin Resistance and Cardiometabolic Diseases: Preclinical and Clinical Evidence. *Reviews on Recent Clinical Trials*.

[6] Jiang H, Guo M, Dong L, Cao C, Wang D, Liang X (2014). Levels of acylation stimulating protein and the complement component 3 precursor are associated with the occurrence and development of coronary heart disease. *Experimental and Therapeutic Medicine*.

[7] van Greevenbroek MMJ, Jacobs M, van der Kallen CJH, Blaak EE, Jansen EHJM, Schalkwijk CG (2012). Human plasma complement C3 is independently associated with coronary heart disease, but only in heavy smokers (the CODAM study). *International Journal of Cardiology*.

[8] King R, Tiede C, Simmons K, Fishwick C, Tomlinson D, Ajjan R (2015). Inhibition of complement C3 and fibrinogen interaction: a potential novel therapeutic target to reduce cardiovascular disease in diabetes. *Lancet (London, England)*.

[9] Széplaki G, Prohászka Z, Duba J, Rugonfalvi-Kiss S, Karádi I, Kókai M (2004). Association of high serum concentration of the third component of complement (C3) with pre-existing severe coronary artery disease and new vascular events in women. *Atherosclerosis*.

[10] Leban N, Jraba K, Chalghoum A, Hassine S, Elhayek D, Denden S (2013). Polymorphism of C3 complement in association with myocardial infarction in a sample of central Tunisia. *Diagnostic Pathology*.

[11] Nagaraj N, Matthews KA, Shields KJ, Barinas-Mitchell E, Budoff MJ, El Khoudary SR (2015). Complement proteins and arterial calcification in middle aged women: Cross-sectional effect of cardiovascular fat. The SWAN Cardiovascular Fat Ancillary Study. *Atherosclerosis*.

[12] Rampidis GP, Benetos G, Benz DC, Giannopoulos AA, Buechel RR (2019). A guide for Gensini Score calculation. *Atherosclerosis*.

[13] Unger T, Borghi C, Charchar F, Khan NA, Poulter NR, Prabhakaran D (2020). 2020 International Society of Hypertension Global Hypertension Practice Guidelines. *Hypertension (Dallas, Tex.: 1979)*.

[14] Li Y, Teng D, Shi X, Qin G, Qin Y, Quan H (2020). Prevalence of diabetes recorded in mainland China using 2018 diagnostic criteria from the American Diabetes Association: national cross sectional study. *BMJ (Clinical Research Ed.)*.

[15] Barrington R, Zhang M, Fischer M, Carroll MC (2001). The role of complement in inflammation and adaptive immunity. *Immunological Reviews*.

[16] Muscari A, Massarelli G, Bastagli L, Poggiopollini G, Tomassetti V, Drago G (2000). Relationship of serum C3 to fasting insulin, risk factors and previous ischaemic events in middle-aged men. *European Heart Journal*.

[17] Phillips CM, Kesse-Guyot E, Ahluwalia N, McManus R, Hercberg S, Lairon D (2012). Dietary fat, abdominal obesity and smoking modulate the relationship between plasma complement component 3 concentrations and metabolic syndrome risk. *Atherosclerosis*.

[18] Nsaiba MJ, Lapointe M, Mabrouk H, Douki W, Gaha L, Pérusse L (2015). C3 Polymorphism Influences Circulating Levels of C3, ASP and Lipids in Schizophrenic Patients. *Neurochemical Research*.

[19] Torres T, Bettencourt N, Mendonça D, Vasconcelos C, Silva BM, Selores M (2014). Complement C3 as a marker of cardiometabolic risk in psoriasis. *Archives of Dermatological Research*.

[20] Garcia-Arguinzonis M, Diaz-Riera E, Peña E, Escate R, Juan-Babot O, Mata P (2021). Alternative C3 Complement System: Lipids and Atherosclerosis. *International Journal of Molecular Sciences*.

[21] Copenhaver MM, Yu CY, Zhou D, Hoffman RP (2020). Relationships of complement components C3 and C4 and their genetics to cardiometabolic risk in healthy, non-Hispanic white adolescents. *Pediatric Research*.

[22] Dissing J, Lund J, Sorensen H (1972). C3 polymorphism in a group of old arteriosclerotic patients. *Human Heredity*.

[23] A Razik N, Y Deep A, Z Abokrisha M, Mosad E, Hasan-Ali H (2022). The relationship between lipid profile after fat loading and coronary artery disease severity assessed by SYNTAX score. *ARYA Atherosclerosis Journal*.

[24] Kiss MG, Binder CJ (2022). The multifaceted impact of complement on atherosclerosis. *Atherosclerosis*.

[25] Chen Y, Ma YT, Yang SJ, Yang YN, Fu ZY, Xie X (2014). Relationship between the acylation-stimulating protein gene and coronary heart disease in the Xinjiang Uygur and Han populations of China. *Genetics and Molecular Research: GMR*.

[26] Ehret GB, Caulfield MJ (2013). Genes for blood pressure: an opportunity to understand hypertension. *European Heart Journal*.

[27] Cai G, Li L, Chen Y, Huang H, Yu L, Xu L (2019). Complement C3 gene polymorphisms are associated with lipid levels, but not the risk of coronary artery disease: a case-control study. *Lipids in Health and Disease*.

[28] Battes LC, Akkerhuis KM, Cheng JM, Garcia-Garcia HM, Oemrawsingh RM, de Boer SPM (2014). Circulating acute phase proteins in relation to extent and composition of coronary atherosclerosis and cardiovascular outcome: results from the ATHEROREMO-IVUS study. *International Journal of Cardiology*.

[29] Császár A, Duba J, Melegh B, Kramer J, Szalai C, Prohászka Z (2001). Increased frequency of the C3*F allele and the Leiden mutation of coagulation factor V in patients with severe coronary heart disease who survived myocardial infarction. *Experimental and Clinical Immunogenetics*.

[30] Golabi P, Kshatriya GK, Kapoor AK (1999). Association of genetic markers with coronary heart disease (myocardial infarction)–a case-control study. *Journal of the Indian Medical Association*.

